# Assessment of variation in immunosuppressive pathway genes reveals *TGFBR2* to be associated with prognosis of estrogen receptor-negative breast cancer after chemotherapy

**DOI:** 10.1186/s13058-015-0522-2

**Published:** 2015-02-10

**Authors:** Jieping Lei, Anja Rudolph, Kirsten B Moysich, Sajjad Rafiq, Sabine Behrens, Ellen L Goode, Paul PD Pharoah, Petra Seibold, Peter A Fasching, Irene L Andrulis, Vessela N Kristensen, Fergus J Couch, Ute Hamann, Maartje J Hooning, Heli Nevanlinna, Ursula Eilber, Manjeet K Bolla, Joe Dennis, Qin Wang, Annika Lindblom, Arto Mannermaa, Diether Lambrechts, Montserrat García-Closas, Per Hall, Georgia Chenevix-Trench, Mitul Shah, Robert Luben, Lothar Haeberle, Arif B Ekici, Matthias W Beckmann, Julia A Knight, Gord Glendon, Sandrine Tchatchou, Grethe I Grenaker Alnæs, Anne-Lise Borresen-Dale, Silje Nord, Janet E Olson, Emily Hallberg, Celine Vachon, Diana Torres, Hans-Ulrich Ulmer, Thomas Rüdiger, Agnes Jager, Carolien HM van Deurzen, Madeleine MA Tilanus-Linthorst, Taru A Muranen, Kristiina Aittomäki, Carl Blomqvist, Sara Margolin, Veli-Matti Kosma, Jaana M Hartikainen, Vesa Kataja, Sigrid Hatse, Hans Wildiers, Ann Smeets, Jonine Figueroa, Stephen J Chanock, Jolanta Lissowska, Jingmei Li, Keith Humphreys, Kelly-Anne Phillips, kConFab Investigators, Sabine Linn, Sten Cornelissen, Sandra Alexandra J van den Broek, Daehee Kang, Ji-Yeob Choi, Sue K Park, Keun-Young Yoo, Chia-Ni Hsiung, Pei-Ei Wu, Ming-Feng Hou, Chen-Yang Shen, Soo Hwang Teo, Nur Aishah Mohd Taib, Cheng Har Yip, Gwo Fuang Ho, Keitaro Matsuo, Hidemi Ito, Hiroji Iwata, Kazuo Tajima, Alison M Dunning, Javier Benitez, Kamila Czene, Lara E Sucheston, Tom Maishman, William J Tapper, Diana Eccles, Douglas F Easton, Marjanka K Schmidt, Jenny Chang-Claude

**Affiliations:** Division of Cancer Epidemiology, German Cancer Research Center (DKFZ), Im Neuenheimer Feld 581, D-69120 Heidelberg, Germany; Department of Cancer Prevention and Control, Roswell Park Cancer Institute, Buffalo, NY 14263 USA; Faculty of Medicine, University of Southampton, University Hospital Southampton, Southampton, SO16 6YD UK; Department of Health Sciences Research, Mayo Clinic, Rochester, MN 55905 USA; Centre for Cancer Genetic Epidemiology, Department of Public Health and Primary Care, University of Cambridge, Cambridge, CB1 8RN UK; Centre for Cancer Genetic Epidemiology, Department of Oncology, University of Cambridge, Worts Causeway, Cambridge, CB1 8RN UK; University Breast Center Franconia, Department of Gynecology and Obstetrics, University Hospital Erlangen, Friedrich-Alexander University Erlangen–Nuremberg, Universitätsstrasse 21-23, 91054 Erlangen, Germany; Division of Hematology and Oncology, Department of Medicine, David Geffen School of Medicine, University of California at Los Angeles, 10833 Le Conte Avenue, Los Angeles, California 90095 USA; Ontario Cancer Genetics Network, Lunenfeld-Tanenbaum Research Institute of Mount Sinai Hospital, 600 University Avenue, Toronto, Ontario M5G 1X5 Canada; Department of Molecular Genetics, University of Toronto, 1 King’s College Circle, Toronto, Ontario M5S 1A8 Canada; Department of Genetics, Institute for Cancer Research, Oslo University Hospital, Radiumhospitalet, Ullernchausseen 70, N-0310 Oslo, Norway; K.G. Jebsen Center for Breast Cancer Research, Institute for Clinical Medicine, Faculty of Medicine, University of Oslo, Kirkeveien 166, 0450 Oslo, Norway; Department of Clinical Molecular Biology (EpiGen), Medical Division, Akershus University Hospital, Sykehusveien 25, 1478 Lørenskog, Norway; Department of Laboratory Medicine and Pathology, Mayo Clinic, Rochester, MN 55905 USA; Division of Molecular Genetics of Breast Cancer, German Cancer Research Center (DKFZ), Im Neuenheimer Feld 580, D-69120 Heidelberg, Germany; Department of Medical Oncology, Erasmus MC Cancer Institute, Groene Hilledijk 301, 3075EA Rotterdam, The Netherlands; Department of Obstetrics and Gynecology, University of Helsinki and Helsinki University Central Hospital, Haartmaninkatu 8, FI-00029 Helsinki, Finland; Department of Molecular Medicine and Surgery, Karolinska Institutet, Stockholm, SE-17177 Sweden; School of Medicine, Institute of Clinical Medicine, Oncology, University of Eastern Finland, Yliopistonranta 1C, FI-70211 Kuopio, Finland; Biocenter Kuopio, Cancer Center of Eastern Finland, University of Eastern Finland, FI-70211 Kuopio, Finland; Cancer Center, Kuopio University Hospital, Puijonlaaksontie 2, 70210 Kuopio, Finland; Vesalius Research Center (VRC), VIB, Herestraat 49, 3000 Leuven, Belgium; Laboratory for Translational Genetics, Department of Oncology, University of Leuven, Herestraat 49, 3000 Leuven, Belgium; Division of Cancer Epidemiology and Genetics, National Cancer Institute, Rockville, MD 20850 USA; Division of Genetics and Epidemiology, The Institute of Cancer Research, Sutton, SM2 5NG UK; Breakthrough Breast Cancer Research Centre, Division of Breast Cancer Research, The Institute of Cancer Research, London, SW3 6JB UK; Department of Medical Epidemiology and Biostatistics, Karolinska Institutet, Box 281, 171 77 Stockholm, Sweden; QIMR Berghofer Medical Research Institute, 300 Herston Road, Brisbane, Queensland 4006 Australia; Institute of Human Genetics, Friedrich Alexander University Erlangen-Nuremberg, Schlossplatz 4, 91054 Erlangen, Germany; Prosserman Centre for Health Research, Lunenfeld-Tanenbaum Research Institute of Mount Sinai Hospital, 600 University Avenue, Toronto, Ontario M5G 1X 5 Canada; Division of Epidemiology, Dalla Lana School of Public Health, University of Toronto, 155 College Street, Toronto, Ontario M5T 3M7 Canada; Lunenfeld-Tanenbaum Research Institute of Mount Sinai Hospital, 600 University Avenue, Toronto, Ontario M5G 1X5 Canada; Institute of Human Genetics, Pontificia Universidad Javeriana, Carrera 7, Bogotá, 11001000 Colombia; Frauenklinik der Stadtklinik Baden-Baden, D-7570 Baden-Baden, Germany; Institute of Pathology, Städtisches Klinikum Karlsruhe, Moltkestrasse 90, 76133 Karlsruhe, Germany; Department of Pathology, Erasmus University Medical Center, 3075EA Rotterdam, The Netherlands; Department of Surgical Oncology, Erasmus MC Cancer Institute, Groene Hilledijk 301, 3075EA Rotterdam, The Netherlands; Department of Clinical Genetics, University of Helsinki and Helsinki University Central Hospital, Haartmaninkatu 8, FI-00029 Helsinki, Finland; Department of Oncology, University of Helsinki and Helsinki University Central Hospital, FI-00029 HUS Helsinki, Finland; Department of Oncology-Pathology, Karolinska Institutet, Stockholm, SE-17177 Sweden; Jyväskylä Central Hospital, Keskussairaalantie 19, 40620 Jyväskylä, Finland; Multidisciplinary Breast Center, University Hospitals Leuven, Herestraat 49, 3000 Leuven, Belgium; Department of Cancer Epidemiology and Prevention, Cancer Center and M Sklodowska-Curie Institute of Oncology, 02-781 Warsaw, Poland; Human Genetics, Genome Institute of Singapore, 60 Biopolis Street 02-01, Singapore, 138672 Singapore; Saw Swee Hock School of Public Health, National University of Singapore, MD3, 16 Medical Drive, Singapore, 117597 Singapore; Centre for Epidemiology and Biostatistics, Melbourne School of Population and Global Health, University of Melbourne, Level 1, 723 Swanston Street, Melbourne, Victoria 3010 Australia; Sir Peter MacCallum Department of Oncology, University of Melbourne, St Andrews Place East, East Melbourne, Victoria 3002 Australia; Division of Molecular Pathology, Netherlands Cancer Institute, Postbus 90203, 1006 BE Amsterdam, The Netherlands; Department of Preventive Medicine, Seoul National University College of Medicine, 103 Daehak-ro, Jongno-gu, Seoul 110-799 Korea; Department of Biomedical Sciences, Seoul National University Graduate School, 1 Gwanak-ro, Gwanak-gu, Seoul 151-742 Korea; Seoul National University College of Medicine, 103 Daehak-ro, Jongno-gu, Seoul 110-799 Korea; Institute of Biomedical Sciences, Academia Sinica, Academia Road Nankang, Taipei, 115 Taiwan; Taiwan Biobank, Academia Sinica, Academia Road Nankang, Taipei, 115 Taiwan; Department of Surgery, Kaohsiung Medical University Chung-Ho Memorial Hospital, No.100 , Tzyou 1st Road, Kaohsiung, 807 Taiwan; College of Public Health, China Medical University, No. 91, Hsueh-Shih Road, Taichung, 40402 Taiwan; Cancer Research Initiatives Foundation, Sime Darby Medical Centre, 1 Jalan SS 12/1A, Subang Jaya, 47500 Selangor Malaysia; Breast Cancer Research Unit, Faculty of Medicine, University Malaya Cancer Research Institute, University Malaya, Lembah Pantai, 59100 Kuala Lumpur, Malaysia; Department of Oncology, Faculty of Medicine, University Malaya, Lembah Pantai, 59100 Kuala Lumpur, Malaysia; Department of Preventive Medicine, Faculty of Medical SciencesLembah Pantai, Kyushu University, Fukuoka, 812-8582 Japan; Division of Epidemiology and Prevention, Aichi Cancer Center Research Institute, 1-1 Kanokoden Chikusa-ku, Nagoya, 464-8681 Aichi Japan; Department of Breast Oncology, Aichi Cancer Center Hospital, 1-1 Kanokoden Chikusa-ku, Nagoya, 464-8681 Aichi Japan; Epidemiology Center for Disease Control and Prevention, Mie University Hospital, 1577 Kurimamachiya-cho, Tsu City, Mie Prefecture 514-8507 Japan; Human Genotyping Unit-Centro Nacional de Genotipado (CEGEN), Human Cancer Genetics Programme, Spanish National Cancer Research Centre (CNIO), 28029 Madrid, Spain; Centro de Investigación en Red de Enfermedades Raras (CIBERER), 46010 Valencia, Spain; Division of Psychosocial Research and Epidemiology, Netherlands Cancer Institute, Postbus 90203, 1006 BE Amsterdam, The Netherlands

## Abstract

**Introduction:**

Tumor lymphocyte infiltration is associated with clinical response to chemotherapy in estrogen receptor (ER) negative breast cancer. To identify variants in immunosuppressive pathway genes associated with prognosis after adjuvant chemotherapy for ER-negative patients, we studied stage I-III invasive breast cancer patients of European ancestry, including 9,334 ER-positive (3,151 treated with chemotherapy) and 2,334 ER-negative patients (1,499 treated with chemotherapy).

**Methods:**

We pooled data from sixteen studies from the Breast Cancer Association Consortium (BCAC), and employed two independent studies for replications. Overall 3,610 single nucleotide polymorphisms (SNPs) in 133 genes were genotyped as part of the Collaborative Oncological Gene-environment Study, in which phenotype and clinical data were collected and harmonized. Multivariable Cox proportional hazard regression was used to assess genetic associations with overall survival (OS) and breast cancer-specific survival (BCSS). Heterogeneity according to chemotherapy or ER status was evaluated with the log-likelihood ratio test.

**Results:**

Three independent SNPs in *TGFBR2* and *IL12B* were associated with OS (*P* <10^−3^) solely in ER-negative patients after chemotherapy (267 events). Poorer OS associated with *TGFBR2* rs1367610 (G > C) (per allele hazard ratio (HR) 1.54 (95% confidence interval (CI) 1.22 to 1.95), *P* = 3.08 × 10^−4^) was not found in ER-negative patients without chemotherapy or ER-positive patients with chemotherapy (*P* for interaction <10^−3^). Two SNPs in *IL12B* (r^2^ = 0.20) showed different associations with ER-negative disease after chemotherapy: rs2546892 (G > A) with poorer OS (HR 1.50 (95% CI 1.21 to 1.86), *P* = 1.81 × 10^−4^), and rs2853694 (A > C) with improved OS (HR 0.73 (95% CI 0.61 to 0.87), *P* = 3.67 × 10^−4^). Similar associations were observed with BCSS. Association with *TGFBR2* rs1367610 but not *IL12B* variants replicated using BCAC Asian samples and the independent Prospective Study of Outcomes in Sporadic versus Hereditary Breast Cancer Study and yielded a combined HR of 1.57 ((95% CI 1.28 to 1.94), *P* = 2.05 × 10^−5^) without study heterogeneity.

**Conclusions:**

*TGFBR2* variants may have prognostic and predictive value in ER-negative breast cancer patients treated with adjuvant chemotherapy. Our findings provide further insights into the development of immunotherapeutic targets for ER-negative breast cancer.

**Electronic supplementary material:**

The online version of this article (doi:10.1186/s13058-015-0522-2) contains supplementary material, which is available to authorized users.

## Introduction

Breast cancer is still the leading cause of cancer-related death in women despite improving survival rates of cancer patients due to earlier detection and expanded treatment options [1], representing nearly 15% of cancer deaths in women [2]. Although at least half of newly diagnosed patients present with early-stage breast cancer, about 20% of these women will experience recurrence at a distant site within 10 years of diagnosis despite chemotherapy and hormonal therapy options [3]. Therefore, limitations of current therapeutic modalities, in particular for estrogen receptor negative (ER-negative) tumors and ER-negative/progesterone receptor (PR-negative) with low expression of human epidermal growth factor receptor 2 (HER2) (triple-negative (TN)) tumors, have led to search for new prognostic tools and therapy targets.

Tumor immunoevasion is recognized as an emerging hallmark of cancer, in addition to the tumor-promoting inflammation [4]. Inhibition of immune response may result from an immunosuppressive state in the tumor microenvironment [5]. Two main types of immune cells involved in the immunosuppression of cancer are the regulatory T cells (Treg cells) and the myeloid derived suppressor cells (MDSCs). Treg cells refer to a subset of T lymphocytes normally expressing CD4 + CD25 + FOXP3+, which play an important role in maintenance of self-tolerance and regulation of immune response [6,7]. MDSCs are a heterogeneous population of immature myeloid cells with expression of CD11b + GR1+ including precursors of macrophages, granulocytes and dendritic cells, which are also involved in tumor immunosuppression [8,9]. Tumor infiltration by immune cells, including Treg cells and MDSCs, has been implicated in cancer patient prognosis after chemotherapy [10-13]. ER-negative tumors typically show higher levels of tumor-infiltrating lymphocytes than ER-positive tumors [14,15]. Indeed, tumor lymphocyte infiltration, including Treg cells has been associated with clinical response to chemotherapy and with prognosis in ER-negative breast cancer [12,13,15], possibly due to the sensitivity of infiltrating lymphocytes to chemotherapeutic agents [16-18].

Therefore, we hypothesized that inherited common variation in genes of the immunosuppressive pathway, including Treg cells and MDSCs, could modulate response to adjuvant chemotherapy, particularly among ER-negative breast cancer patients. We evaluated genetic associations of single nucleotide polymorphisms (SNPs) located in or near (within 50 kb upstream and downstream) 133 candidate genes of the immunosuppressive pathway with overall survival (OS) and breast cancer-specific survival (BCSS) in breast cancer patients of European ancestry from 16 Breast Cancer Association Consortium (BCAC) studies [19] and performed replications for the variants with the strongest associations using two independent patient samples.

## Methods

### Study sample

We selected women of European ancestry diagnosed with histologically verified primary invasive but not metastatic breast cancer (stage I to III disease) and restricted to women with available age information, because age is an important risk factor for breast cancer (flow chart of patient selection in Additional file 1: Figure S1). The cause of death for an individual patient was recorded by hospital, cancer registry or health offices in the respective studies. The majority of the studies were all carried out in developed countries where deaths were accurately and mandatorily reported and causes of death had to be reported by the physicians, thus, the vast majority of deaths were reliably captured. Follow up was censored at 10 years from study entry. Studies with fewer than 10 events for all-cause mortality within this period were excluded as well as women with missing information on ER status, adjuvant chemotherapy, vital status and cause of death. Excluded patients had a similar mean age as compared to patients included in the study (55.7 versus 56.8 years), had more family history of breast cancer (30.53% versus 23.01%), had a lower frequency in receiving adjuvant chemotherapy (30.56% versus 39.85%), and had similar distribution in tumor stage, grade, size, and ER/PR/HER2 status. A total of 11,668 patients (9,334 with ER-positive disease, 2,334 with ER-negative disease) from 16 studies in BCAC were included (Additional file 2: Table S1a). Of these patients, 4,650 patients (3,151 with ER-positive disease and 1,499 with ER-negative disease) had received adjuvant chemotherapy, 7,018 patients (6,183 with ER-positive disease and 835 with ER-negative disease) did not receive chemotherapy.

For the replication analyses in ER-negative patients who had received adjuvant chemotherapy, we used four Asian studies in BCAC as one sample set and the Prospective Study of Outcomes in Sporadic versus Hereditary breast cancer (POSH) study (consisting of early-onset patients of European ancestry) as a second sample set [20,21]. As for the discovery, we included only ER-negative patients treated with adjuvant chemotherapy and restricted follow-up to 10 years after diagnosis. Thus, 372 breast cancer patients (42 events) from the BCAC Asian studies and 127 early-onset breast cancer patients (62 events) in the POSH study were included (Additional file 2: Table S1b). All studies were approved by the relevant ethics committees and all participants had signed an informed consent (Additional file 2: Table S1a and S1b).

### SNP selection and genotyping

Genes related to Treg cell and MDSC pathways were identified through an extensive and comprehensive literature review in PubMed [22-34], using the search terms immunosuppression/immunosuppressive, regulatory T cells/Treg cells/FOXP3+ T cells, myeloid derived suppressor cells/MDSCs, immunosurveillance, and tumor escape, as only the broader immune pathways were accessible in the KEGG [35] and GO [36] databases. The final candidate gene list included 133 immunosuppression-related genes (Additional file 2: Table S2). SNPs with minor allele frequency (MAF) >0.05 within 50 kb upstream and downstream of each gene were identified using HapMap CEU genotype data and dbSNP 126 as references [37].

For the BCAC studies, study samples were genotyped for 211,155 SNPs using a custom Illumina iSelect array (iCOGS) designed for the Collaborative Oncological Gene-Environment Study (COGS) [19]. Of the 211,155 SNPs, 4,246 SNPs were located in the candidate genes within a window of ±50 kb. A series of centralized quality controls after genotyping led to exclusion of 243 SNPs. The exclusion criteria included a called rate <95% in all samples genotyped with iCOGS; being monomorphic; deviation from Hardy-Weinberg equilibrium (HWE) with a *P*-value <10^−7^, and concordance in duplicate samples <98%. After restricting the study sample to the subjects eligible (n = 11,668), we additionally excluded 393 SNPs with MAF <0.05 and deviation from HWE (*P*-value <10^−7^). A total of 3,610 SNPs passed all quality controls and were analyzed.

We used imputed genotype data of the POSH study. Imputation of POSH genome-wide association study (GWAS) data (genotyped using the Illumina 660-Quad SNP array, San Diego, CA, USA) was performed utilizing MACH 1.0 [38] based on the CEU population from HapMap phase 2 [37] and a posterior probability of 0.9. Imputation data were excluded based on MAF <0.01 and HWE with *P*-value <10^−4^. More details of POSH data are described elsewhere [39].

### Statistical methods

Cox proportional hazard regression analysis with right truncation at 10 years after diagnosis was applied to model patient survival. Each single SNP was assessed as an ordinal variable (coded as 0, 1 and 2 respectively, according to number of minor allele). Analyses were adjusted for age at diagnosis and nine principal components to account for population substructure and stratified by study. To account for possible confounding due to differences in patient characteristics, we included tumor size, tumor grade and node status as further covariates. Delayed entry (left truncation) was used to reduce potential survival bias due to eligible patients who died before recruitment into the study or before the blood draw. Follow-up time was thus calculated from the date of interview or blood draw until event or censoring (date of last follow up). To determine the number of independent SNPs for adjustment of multiple testing, we applied the option, --indep-pairwise, in PLINK [40]. SNPs were pruned by linkage disequilibrium (LD) of *r*^2^ < 0.2 for a window size of 50 SNPs and step size of 10, yielding 699 independent SNPs. The significance threshold using Bonferroni correction corresponding to an alpha of 5% had a *P-*value <7.15 × 10^−5^.

In the primary analysis, we modeled OS in a multivariate Cox proportional hazard regression framework for ER-negative breast cancer patients separately for those who received adjuvant chemotherapy and those who did not receive adjuvant chemotherapy. To investigate whether SNP associations were restricted to ER-negative breast cancer, we assessed heterogeneity of associations between these two subgroups by using interaction terms between chemotherapy and SNPs, which were evaluated using likelihood ratio tests, comparing models with and without the interaction term. We also assessed whether selected SNPs associated with OS in ER-negative breast cancer patients who received adjuvant chemotherapy were associated with OS in ER-positive breast cancer patients treated with chemotherapy. Possible heterogeneity in the associations of SNPs with OS for patients who received chemotherapy according to ER status was assessed statistically by using interaction terms between ER status and SNPs and evaluated using likelihood ratio tests. In secondary analysis, we evaluated SNP associations with OS separately for ER-negative/PR-negative and TN breast cancer patients who received or did not receive adjuvant chemotherapy, respectively. Additionally, we assessed the associations of the SNPs with breast cancer-specific survival (BCSS) in ER-negative patients who received adjuvant chemotherapy. All statistical tests mentioned above were two-sided and conducted using SAS 9.2 (Cary, NC, USA).

For genes with multiple associated SNPs, HaploView was used to examine LD between SNPs. To identify potentially independently associated SNPs, we ran models including multiple associated SNPs within a gene. The proportional hazard assumption for the associated SNPs was assessed according to Grambsch and Therneau [41] and no significant deviation was noted. Cluster plots for the most significant SNPs were examined among BCAC samples and all showed good discrimination of three genotypes.

Meta-analyses were performed to summarize the results from the discovery and replication studies and to determine study heterogeneity using the *I*^2^ index and *Q*-statistics [42,43] and forest plots were generated using R (version 2.15.2).

## Results

A descriptive summary of characteristics of the study population with available follow-up information is given in Table 1. There were 9,334 ER-positive breast cancer patients and 2,334 ER-negative breast cancer patients, of whom 1,904 had ER-negative/PR-negative disease and 1,007 TN disease. Of patients who had received adjuvant chemotherapy, 3,151 had ER-positive disease (376 events), 1,499 ER-negative disease (267 events), 1,271 ER-negative/PR-negative disease (221 events) and 692 TN disease (111 events).

**Table 1:** **Characteristics of the BCAC European study participants**

**Characteristics**	**ER-negative patients who received chemotherapy**	**Percent**	**ER-negative patients who did not receive chemotherapy**	**Percent**	**ER-positive patients who received chemotherapy**	**Percent**
Number of patients	1499	100.00	835	100.00	3151	100.00
Age at diagnosis (mean ± SD, years)	51.69 ± 10.85		59.46 ± 12.18		51.74 ± 9.88	
Family history						
No	974	64.98	457	54.73	2233	70.87
Yes	275	18.35	148	17.72	596	18.91
Missing	250	16.68	230	27.54	322	10.22
Tumor stage						
1	356	23.75	401	48.02	606	19.23
2	804	53.64	259	31.02	1751	55.57
3	182	12.14	58	6.95	526	16.69
Missing	157	10.47	117	14.01	268	8.51
Histological grade						
Well-differentiated	23	1.53	97	11.62	390	12.38
Moderately differentiated	293	19.55	310	37.13	1624	51.54
Poorly/undifferentiated	1183	78.92	428	51.26	1137	36.08
Tumor size						
≤2 cm	664	44.30	528	63.23	1387	44.02
≥2 cm to ≤5 cm	744	49.63	271	32.46	1490	47.29
≥5 cm	91	6.07	36	4.31	274	8.70
Lymph node status						
Negative	735	49.03	651	77.96	976	30.97
Positive	764	50.97	184	22.04	2175	69.03
PR status						
PR-negative	1271	84.79	633	75.81	546	17.33
PR-negative HER2-negative	692	46.16	315	37.72	304	9.65

Results are presented as number of patients unless stated otherwise. BCAC, Breast Cancer Association Consortium; ER, estrogen receptor; PR, progesterone receptor; SD, standard deviation; HER2, human epidermal growth factor receptor 2.

**Figure 1:**
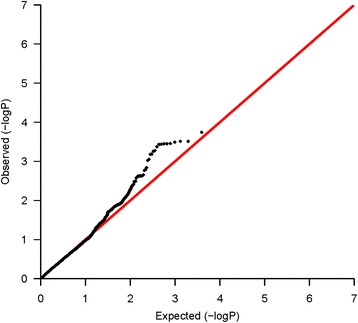
**Quantile-quantile (QQ) plot of the observed** ***P*** **-values for associations with overall survival in estrogen receptor (ER)-negative patients who received chemotherapy.** QQ plot shows the observed -log10 *P*-values (y axis) versus the expected -log10 *P*-values (x axis) for association of 3,610 SNPs in the immunosuppression pathway, with overall survival in ER-negative breast cancer patients who received adjuvant chemotherapy. The black dots indicate that there is inflation for observed associations.

A quantile-quantile (QQ) plot for tests of associations with OS for the 3,610 evaluated SNPs in ER-negative breast cancer patients who received adjuvant chemotherapy is shown in Figure 1. Three independent genetic variants in the two genes, *TGFBR2* and *IL12B*, showed associations with OS (*P* <10^−3^) only in ER-negative breast cancer patients who received adjuvant chemotherapy. None of the associations was significant after Bonferroni correction (*P* <7.15 × 10^−5^) (Table 2). In ER-negative breast cancer patients who did not receive chemotherapy, none of the SNPs were associated (*P* <10^−3^). The results for all assessed 3,610 SNPs in ER-negative breast cancer patients treated with adjuvant chemotherapy are summarized in Additional file 2: Table S3.

**Table 2:** ***TGFBR2*** **and** ***IL12B*** **SNPs associated with overall survival (** ***P*** **-value <0.001) in ER-negative patients with chemotherapy**

**Chr**	**Gene**	**SNP**	**Minor allele**	**MAF**	**ER-negative patients who received adjuvant chemotherapy**				**ER-negative patients who did not receive adjuvant chemotherapy**					**ER-positive patients who received adjuvant chemotherapy**				
					**Cases, number**	**Events, number**	**HR** ^**a**^ **(95% CI)**	***P*** **-value**	**Cases, number**	**Events, number**	**HR** ^**a**^ **(95% CI)**	***P*** **-value**	***P*** **heterogeneity** ^**b**^	**Cases, number**	**Events, number**	**HR** ^**a**^ **(95% CI)**	***P*** **-value**	***P*** **heterogeneity** ^**c**^
3	*TGFBR2*	rs1367610	C	0.14	1499	267	1.54 (1.22, 1.95)	3.08 × 10^−4^	834	155	0.78 (0.55, 1.13)	0.191	8.82 × 10^−4^	3151	376	0.88 (0.70, 1.10)	0.251	2.62 × 10^−4^
5	*IL12B*	rs2546892	A	0.17	1499	267	1.50 (1.21, 1.86)	1.81 × 10^−4^	835	155	0.99 (0.74, 1.33)	0.968	0.025	3151	376	0.99 (0.82, 1.20)	0.940	4.63 × 10^−3^
5	*IL12B*	rs2853694	C	0.51	1499	267	0.73 (0.61, 0.87)	3.67 × 10^−4^	835	155	1.06 (0.85, 1.33)	0.596	0.020	3151	376	0.95 (0.82, 1.10)	0.529	0.023

^a^HR adjusted for age of diagnosis, tumor size, tumor grade, node status and nine principal components to account for population substructure and stratified by study. ^b^ *P-*value for test of heterogeneity between ER-negative breast cancer patients who received adjuvant chemotherapy and ER-negative breast cancer patients who did not receive adjuvant chemotherapy. ^c^ *P-*value for test of heterogeneity between ER-negative breast cancer patients who received adjuvant chemotherapy and ER-positive breast cancer patients who received adjuvant chemotherapy. TGFBR2, transforming growth factor, beta receptor II; SNP, single nucleotide polymorphism; ER, estrogen receptor; Chr, chromosome; MAF, minor allele frequency; HR, hazard ratio; IL12B, interleukin 12B; and CI, confidence interval.

**Figure 2:**
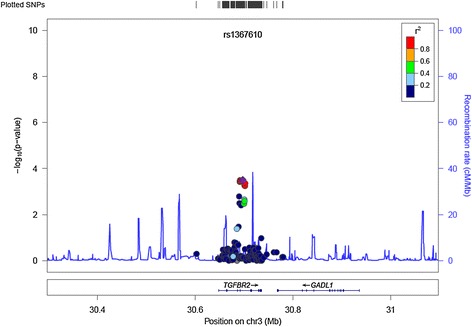
**Regional association plot for single nucleotide polymorphisms (SNPs) in** ***TGFBR2.*** The regional plot shows associations with overall survival in ER-negative breast cancer patients who received adjuvant chemotherapy, for all SNPs in *TGFBR2*. The y-axis shows the -log10 *P*-value. The purple diamond indicates SNP rs1367610, with the most significant association in *TGFBR2*. Chr, chromosome.

**Figure 3:**
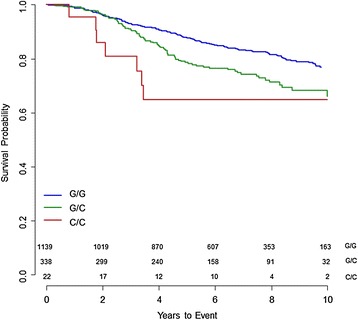
**Kaplan-Meier survival curves of overall survival in estrogen receptor (ER)-negative patients who had chemotherapy for** ***TGFBR2*** **rs1367610.** The survival curves for *TGFBR2* rs1367610 (G > C) stratified by genotype are shown for the Breast Cancer Association Consortium European sample. The *P*-value of the log-rank test was 2.0 × 10^−4^. The number of events and cases in parenthesis for each genotype are GG (180/1139, blue line), GC (80/338, green line) and CC (7/22, red line) respectively.

In *TGFBR2,* the strongest association in ER-negative patients who received chemotherapy was seen for SNP rs1367610 (G > C) (per allele hazard ratio (HR) 1.54 (95% confidence interval (CI) 1.22, 1.95), *P* = 3.08 × 10^−4^). A regional association plot for all SNPs in *TGFBR2* is shown in Figure 2. The Kaplan-Meier survival curve stratified by genotype of SNP rs1367610 is shown in Figure 3. For the univariate survival curves, the *P*-value of the log-rank test was 2.0 × 10^−4^. There was no evidence of heterogeneity for the association across eight studies with at least ten events in ER-negative patients with chemotherapy (Additional file 1: Figure S2). This SNP was not associated with OS in ER-negative patients who did not receive chemotherapy (*P*-value for interaction = 8.82 × 10^−4^) or with ER-positive patients who received chemotherapy (*P*-value for interaction = 2.62 × 10^−4^). Variant alleles of nine further SNPs in *TGFBR2* in strong LD with rs1367610 (*r*^2^ ≥ 0.97) were similarly associated with poorer OS in ER-negative breast cancer patients treated with chemotherapy (Additional file 2: Table S3). After accounting for rs1367610, none of other nine *TGFBR2* variants showed association with OS.

Two independent SNPs in *IL12B* (*r*^2^ = 0.20) showed associations with ER-negative disease after chemotherapy: rs2546892 (G > A) with poorer OS (HR 1.50 (95% CI 1.21, 1.86), *P* = 1.81 × 10^−4^), and rs2853694 (A > C) with improved OS (HR 0.73 (95% CI 0.61, 0.87), *P* = 3.67 × 10^−4^). These SNPs were not associated with OS in ER-negative patients who did not receive chemotherapy (*P*-value for interaction: 2.53 × 10^−2^ for rs2546892, 1.98 × 10^−2^ for rs2853694), or in ER-positive patients who received chemotherapy (*P*-value for interaction: 4.63 × 10^−3^ for rs2546892, 2.27 × 10^−2^ for rs2853694) (Table 2). Three other SNPs in *IL12B* (rs2853697, rs2569254 and rs3181225) in high LD with rs2546892 (*r*^2^ ≥ 0.81, Additional file 1: Figure S3a) were also associated with OS (*P* <10^−3^) (Additional file 2: Table S3). After adjusting for rs2853694, rs2546892 (but not the other three SNPs) was still associated with OS, suggesting that there are two potential independently associated variants in *IL12B.*

In the secondary analysis of ER-negative/PR-negative patients who received chemotherapy, rs1488369 (A > G) in a further gene, *CCR9,* was observed to be associated with an improved OS (HR 0.72 (95% CI 0.59, 0.87), *P* = 8.63 × 10^−4^), besides SNPs in *TGFBR2* and *IL12B* (Additional file 2: Table S4). An association was not found for ER-negative PR-negative patients without chemotherapy (*P*-value for interaction = 1.78 × 10^−2^). This SNP was associated (HR 0.75 (95% CI 0.63, 0.90, *P* = 1.70 × 10^−3^) in patients with ER-negative disease (Additional file 2: Table S3).

In TN breast cancer patients treated with chemotherapy, rs2285440 (A > C), rs1726599 (C > A) and rs6956139 (C > A) in moderate LD (*r*^2^ ≥ 0.38) located in *HDAC9* showed associations with two-fold increased HRs (HR 1.92 to 2.41, *P* <10^−3^) (Additional file 1: Figure S3b, Additional file 2: Table S4). None of the three SNPs was associated with OS in TN patients who did not receive chemotherapy. SNP rs2285440 remained strongly associated (HR 2.09 (95% CI 1.06, 4.15) after adjusting for the other two SNPs. This SNP showed a weaker association in patients with ER-negative disease (HR 1.47 (95% CI 1.09, 1.98), *P* = 1.26 × 10^−2^) (Additional file 2: Table S3).

Additionally, rs9863120 (A > G) located in *EIF2A* was associated with a significantly improved OS in TN patients who received chemotherapy (per allele HR 0.53 (95% CI 0.38, 0.74), *P* = 1.87 × 10^−4^) but not those without chemotherapy (*P*-value for interaction = 8.02 × 10^−4^) (Additional file 2: Table S4). In patients with ER-negative disease, this SNP showed a weaker association (HR 0.78 (95% CI 0.65, 0.95), *P* = 1.43 × 10^−2^) (Additional file 2: Table S3).

We also assessed the associations of the immunosuppressive pathway SNPs with BCSS among ER-negative breast cancer patients who received chemotherapy. The results were in line with the findings of the OS analysis. The most strongly associated SNP rs1872987 in *TGFBR2* with BCSS is in high LD with rs1367610 (*r*^2^ = 0.99, HR 1.69 (95% CI 1.31, 2.19), *P* = 6.26 × 10^−5^) and the same SNPs in *IL12B* were found to be associated. Additionally, an SNP, rs658230 (G > A) in *PRKCQ*, was associated with an improved BCSS (per allele HR 0.70 (95% CI 0.57, 0.86), *P* = 6.95 × 10^−4^) and an SNP rs9579165 (A > G) in *FLT3* showed a poorer BCSS (per allele HR 1.87 (95% CI 1.30, 2.70), *P* = 7.73 × 10^−4^) (Additional file 2: Table S5).

We performed two independent replications for the SNPs in *TGFBR2* and *IL12B* specifically associated with OS only in the ER-negative breast cancer patients treated with adjuvant chemotherapy using the BCAC Asian samples and the (European) POSH study. The association of *TGFBR2* rs1367610 with OS in ER-negative patients after adjuvant chemotherapy was replicated in both BCAC Asian samples (HR 2.18 (95% CI 0.85, 5.60), *P* = 1.05 × 10^−1^) as well as in the POSH study (HR 1.59 (95% CI 0.94, 2.69), *P* = 8.39 × 10^−2^), and was significant (HR 1.71 (95% CI 1.08, 2.72) in the replication samples combined. *IL12B* rs2853694 and rs2546892 did not replicate in the two studies. Meta-analysis of the discovery and replication studies yielded for *TGFBR2* rs1367610 an HR of 1.57 (95% CI 1.28, 1.94, *P* = 2.05 × 10^−5^) without evidence of heterogeneity (*I*^2^ = 0%; *P* heterogeneity = 0.78) (Table 3).

**Table 3:** **Associations of** ***TGFBR2*** **and** ***IL12B*** **SNPs with overall survival in discovery and replication samples**

**Breast cancer patients**	***TGFBR2*** **rs1367610 (G > C)**				***IL12B*** **rs2546892 (G > A )**				***IL12B*** **rs2853694 (A > C)**			
	**Cases, number**	**Events, number**	**HR (95% CI)**	***P*** **-value**	**Cases, number**	**Events, number**	**HR (95% CI)**	***P*** **-value**	**Cases, number**	**Events, number**	**HR (95% CI)**	***P*** **-value**
Discovery												
ER-negative and received chemotherapy^a^	1499	267	1.54 (1.22, 1.95)	3.08 × 10^−4^	1499	267	1.50 (1.21, 1.86)	1.81 × 10^−4^	1499	267	0.73 (0.61, 0.87)	3.67 × 10^−4^
	*I* ^2c^ = 86.7%; *P* heterogeneity^d^ = 5.00 × 10^−4^				*I* ^2c^ = 78.5%; *P* heterogeneity^d^ = 9.50 × 10^−3^				*I* ^2c^ = 75.9%; *P* heterogeneity^d^ = 0.016			
Replication												
ER-negative and received chemotherapy												
BCAC Asian studies^a^	372	42	2.18 (0.85, 5.60)	0.105	372	42	0.62 (0.30, 1.26)	0.187	372	42	1.03 (0.63, 1.67)	0.919
POSH study^b^	127	62	1.59 (0.94, 2.69)	0.084	127	62	1.09 (0.67, 1.78)	0.715	127	62	0.87 (0.62, 1.22)	0.408
Combined replication												
ER-negative and received chemotherapy	499	104	1.71 (1.08, 2.72)	0.022	499	104	0.91 (0.61, 1.36)	0.659	499	104	0.92 (0.69, 1.21)	0.535
	*I* ^2c^ = 0%; *p* heterogeneity^d^ = 0.567				*I* ^2c^ = 40.4%; *P* heterogeneity^d^ = 0.20				*I* ^2c^ = 0%; *P* heterogeneity^d^ = 0.577			
Combined overall												
ER-negative received adjuvant chemotherapy	1998	371	1.57 (1.28, 1.94)	2.05 × 10^−5^	1998	371	1.11 (0.70, 1.76)	0.653	1998	371	0.78 (0.67, 0.90)	8.00 × 10^−4^
	*I* ^2c^ = 0%; *P* heterogeneity^d^ = 0.781				*I* ^2c^ = 68.3%; *P* heterogeneity^d^ = 0.04				*I* ^2c^ = 6.4%; *P* heterogeneity^d^ = 0.344			

^a^HR adjusted for age of diagnosis, tumor size, tumor grade, node status and principal components to account for population substructure and stratified by study. ^b^HR adjusted for age of diagnosis, tumor size, tumor grade, node status and metastasis status. ^c^ *I* ^2^ index derived on the basis of effect estimate and variance in each study. ^d^ *P-*value for test of heterogeneity between studies using the DerSimonian-Laird test. TGFBR2, transforming growth factor, beta receptor II; SNP, single nucleotide polymorphism; HR, hazard ratio; ER, estrogen receptor; BCAC, Breast Cancer Association Consortium; IL12B, interleukin 12B; CI, confidence interval; and POSH, Prospective Study of Outcomes in Sporadic versus Hereditary Breast Cancer.

## Discussion

In this study, we found that common variants in *TGFBR2* have prognostic value for ER-negative breast cancer patients who received adjuvant chemotherapy. Our hypothesis was confirmed that this was specific for ER-negative disease, as the *TGFBR2* variants were clearly not associated with OS in ER-positive breast cancer patients who received chemotherapy. The *TGFBR2* variants also have predictive value, as the association with OS in ER-negative breast cancer patients was significantly differential according to treatment with chemotherapy.

*TGFBR2* (3p22) encodes the transforming growth factor beta (TGF-β) receptor II, which is a transmembrane serine/threonine protein kinase receptor in the TGF-β signaling pathway [44]. As an important cytokine in tumor microenvironment, TGF-β has been considered to have a dual role in tumor suppression at early stages but then later promoting tumor invasion and metastasis [44,45]. Specifically, TGF-β functions as a stimulator in the tumor microenvironment to promote Treg cell proliferation and immune evasion [46]. An ER-negative tumor is normally associated with a higher level of infiltrating lymphocytes [14,15]. TGF-β receptor II plays a key role in the TGF-β signaling pathway, as all three TGF-β isoforms bind to this receptor [45]. Early genetic loss of *TGFBR2* may lead to rapid tumor growth [45]. *TGFBR2* has been identified as a susceptibility locus for breast cancer risk [19] and its expression in cancer-associated fibroblasts was found to be a prognostic marker for pre-menopausal breast cancer [47]. Since the immune-modulatory activities of TGF-ß have implications for many diseases, many drugs targeting the TGF-ß signaling have been developed. Based on our findings, it is conceivable that *TGFBR2* variants may have prognostic and predictive value also for the outcome of TGF-ß signal inhibition.

*TGFBR2* rs1367610 was recently reported to be possibly associated with BCSS in ER-negative patients treated with adjuvant chemotherapy using the COGS samples, however, replication in independent studies was not carried out [48]. The prior COGS study examined associations with breast cancer survival for 7,020 SNPs in 557 genes related to immune response and inflammation [48]. There were about 70 genes (1,694 SNPs) that overlapped between the two studies. The discovery sample of our study is somewhat smaller due to restriction to early breast cancer (stage I to III disease) and truncation of follow up to 10 years to minimize the influence of comorbidity on survival. However, we confirmed the prognostic value of *TGFBR2* in the independent POSH study as well as in the Asian samples without study heterogeneity and also showed *TGFBR2* variants to be related to both OS and BCSS.

All the top SNPs (*P*-value <10^−3^) in *TGFBR2* were in the intron of this gene. According to the UCSC genome browser, the best-hit rs1367610 is located in the transcription factor binding site, and rs1019856, rs1841528 and rs6550007 are in both the DNase I hypersensitivity clusters and transcription factor binding sites. In addition, from the HaploReg online tool, we found that rs6550007 (*r*^2^ = 0.98 with rs1367610) may change the binding site of forkhead box P3 (Foxp3), which is an important transcription factor and a typical surface marker of Treg cells (Additional file 1: Figure S4). The top *TGFBR2* SNPs associated with breast cancer OS are not included in the GeneVar gene expression variation database [49]. They lay in a different LD block from that of the reported breast cancer risk-associated SNPs that led to the identification of *TGFBR2* as a breast cancer susceptibility locus [19]. Neither rs1367610 nor SNPs in high LD was associated with breast cancer risk in the BCAC studies. It would be worth looking for potential regulatory SNPs further than 50 kb away, and further functional analyses are necessary to identify the causal variant.

Although *IL12B* was found to be associated with OS and with BCSS, also reported as possibly associated in the previous publication [48], we were not able to replicate this finding using the two studies, which were smaller than the discovery sample. If a real association was overestimated in the discovery sample, a much larger study sample would be required for replication. *IL12B* (5q31.1-q33.1) encodes IL12 p40, which acts as a subunit of the heterodimeric structure of cytokine IL12 and IL23, two important immune cytokines in cell-mediated immunity [50,51]. IL12 and IL23 can separately promote naïve T cells into T helper (Th)1 cells and Th17 cells *in vivo* [51], and the balance between Th17 cells and Treg cells is a key factor in maintaining a normal immune response [52].

Further genes, *LZTFL1/CCR9*, *HDAC9* and *EIF2A,* as well as *PRKCQ* and *FLT3*, were implicated to play a role in OS and/or BCSS for ER-negative patients after chemotherapy. These findings warrant follow up in large patient samples, because more variants in immunosuppressive pathway genes are potentially associated with prognosis of breast cancer.

Three GWAS studies to date have been carried out to investigate inherited genetic variants associated with overall or breast cancer-specific mortality of breast cancer [39,53,54]. In part due to the moderate study size involved, few associations have been identified and confirmed. On the other hand, a GWAS of clinical outcome in breast cancer patients who received adjuvant tamoxifen therapy identified a new locus associated with recurrence-free survival [55]. Therefore, germ-line genetic variation associated with breast cancer prognosis may be more easily detected when considering specific treatment subgroups and/or cancer subtypes.

The main strengths of this study include the uniform genotyping procedures, stringent centralized quality controls and large sample size, which provides us with sufficient statistical power to detect associations between genetic variants with moderate effects and breast cancer prognosis. The availability of centrally collated and harmonized information on molecular subtype, clinical treatment, and follow up in BCAC allowed us to assess potential differential SNP associations according to chemotherapy and also according to ER status. We used the iCOGS array with 3,610 SNPs to comprehensively assess these pathways. However, tagging SNP coverage varied across different candidate genes and could not capture variation entirely across all of the immunosuppressive pathway-related loci. Only genotyped data but not imputed data were used. As we focused on single SNP assessment and did not perform multi-marker analyses, we might have not captured all truly associated loci. Two independent study samples were employed to replicate the most promising findings. The replication in the Asian population, a different ethnic group, also suggests that the observed association with *TGFBR2* variants is likely to be real. However, further genetic and functional studies are still required to identify the causal variants and the mechanisms underlying the associations observed in this study.

## Conclusions

Our findings indicate that elucidating genetic variants, which influence inhibition of tumor immunity, may provide prognostic and predictive markers of chemotherapy for ER-negative breast cancer and could lead to further therapy targets.

## Additional files

Additional file 1: Figure S1. Flow chart of patient selection. Figure S2 Forest plot of eight studies with at least ten events for TGFBR2 rs1367610. Figure S3a Linkage disequilibrium of five top single nucleotide polymorphisms (SNPs) in IL12B associated with estrogen receptor (ER)-negative patients with chemotherapy. Figure S3b linkage disequilibrium of seven top SNPs in HDAC9 associated with triple-negative patients with chemotherapy. Figure S4 UCSC browser graphic for TGFBR2 rs1367610. Additional file 2: Table S1. Description of studies included: (S1a) Sixteen Breast Cancer Association Consortium (BCAC) European studies in the discovery analysis; (S1b) Four BCAC Asian studies and Prospective Study of Outcomes in Sporadic versus Hereditary Breast Cancer (POSH) study in the replication analysis. Table S2 List of 133 candidate genes related to the immunosuppressive pathway by chromosomal position. Table S3 Associations of 3610 single nucleotide polymorphisms (SNPs) in the immunosuppressive pathway with overall survival in estrogen receptor (ER)-negative patients with chemotherapy. Table S4 Top SNPs associated with overall survival of ER-negative/progesterone receptor (PR)-negative and triple-negative patients who received chemotherapy. Table S5 Top SNPs associated with breast cancer-specific survival of ER-negative patients who received chemotherapy.

## Abbreviations

BCAC: Breast Cancer Association Consortium
BCSS: breast cancer-specific survival
CCR9: chemokine (C-C motif) receptor 9
CI: confidence interval
COGS: Collaborative Oncological Gene-Environment Study
EIF2A: eukaryotic translation initiation factor 2 alpha
ER: estrogen receptor
FLT3: fms-related tyrosine kinase 3
Foxp3: forkhead box P3
GWAS: genome-wide association studies
HDAC9: histone deacetylase 9
HER2: human epidermal growth factor receptor 2
HR: hazard ratio
HWE: Hardy-Weinberg equilibrium
IL12B: interleukin 12B
LD: linkage disequilibrium
LZTFL1: leucine zipper transcription factor-like 1
MAF: minor allele frequency
MDSCs: myeloid derived suppressor cells
OS: overall survival
POSH: Prospective Study of Outcomes in Sporadic versus Hereditary Breast Cancer Study
PR: progesterone receptor
PRKCQ: protein kinase C, theta
QQ: quantile-quantile
SD: standard deviation
SNP: single nucleotide polymorphism
TGFBR2: transforming growth factor, beta receptor II
TGF-β: transforming growth factor beta
Th: T helper
TN: triple negative
Treg cells: regulatory T cells
