# Supplementary figures and images for: Assessment of variation in immunosuppressive pathway genes reveals TGFBR2 to be associated with prognosis of estrogen receptor-negative breast cancer after chemotherapy
Source: Breast Cancer Res. 2015 Feb 10;17(1):18. doi: 10.1186/s13058-015-0522-2 (PMC4374346; doi:10.1186/s13058-015-0522-2)

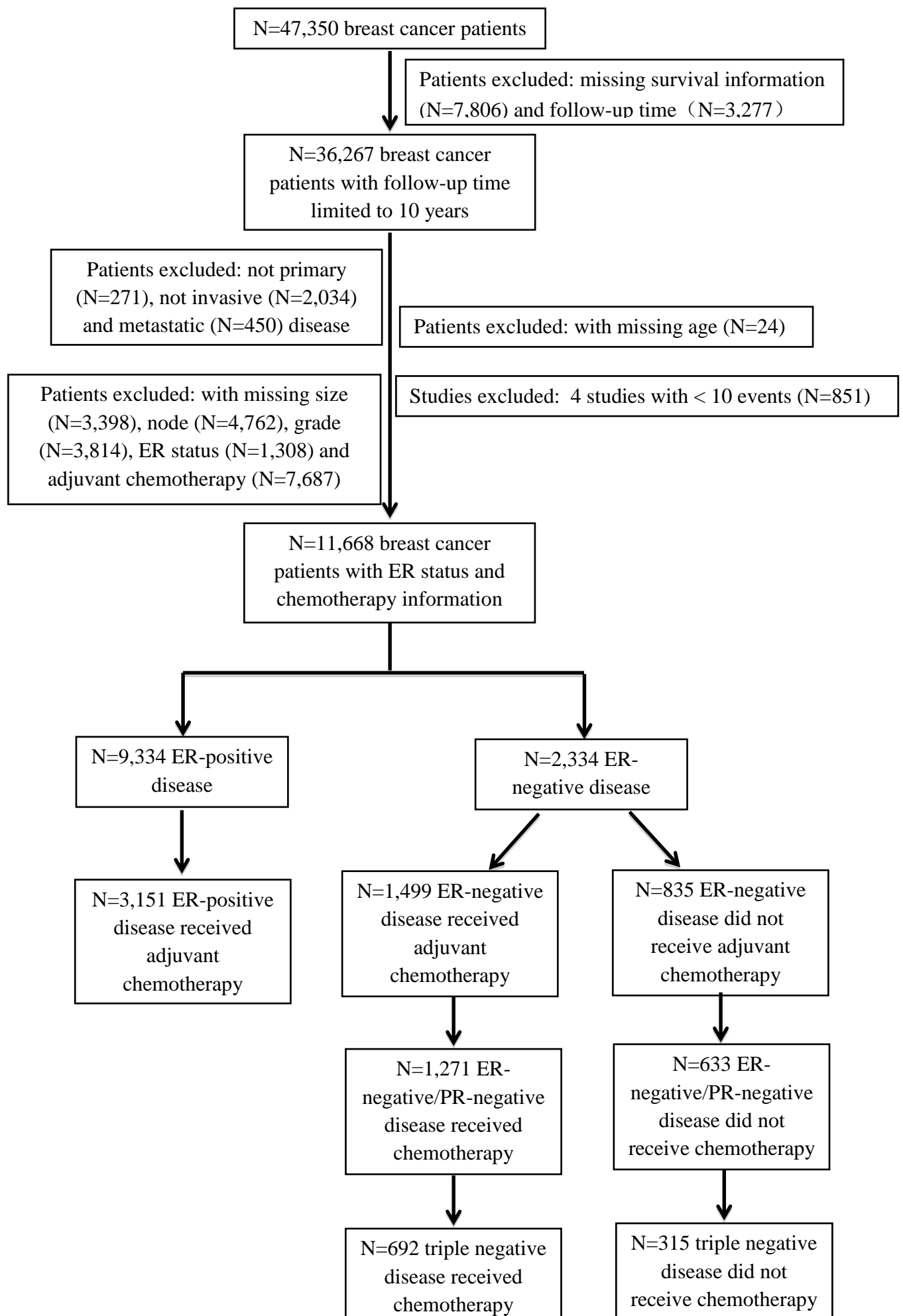

Supplement: Additional file 1: Figure S1. — Flow chart of patient selection. Figure S2 Forest plot of eight studies with at least ten events for TGFBR2 rs1367610. Figure S3a Linkage disequilibrium of five top single nucleotide polymorphisms (SNPs) in IL12B associated with estrogen receptor (ER)-negative patients with chemotherapy. Figure S3b linkage disequilibrium of seven top SNPs in HDAC9 associated with triple-negative patients with chemotherapy. Figure S4 UCSC browser graphic for TGFBR2 rs1367610. [file 13058_2015_522_MOESM1_ESM.zip › 4888551391406903_add1/Figure S1.pdf]

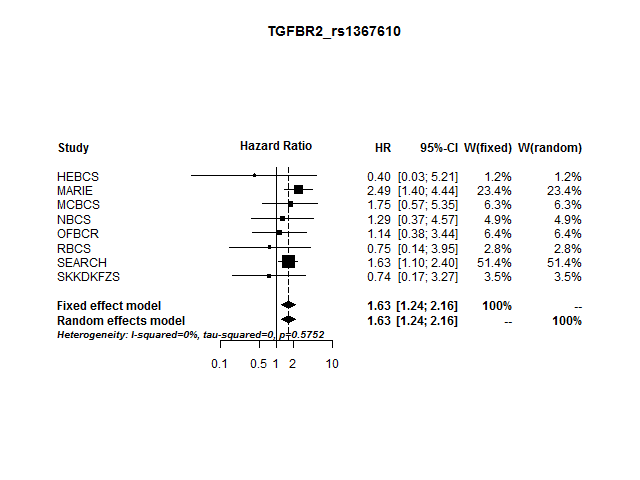

Supplement: Additional file 1: Figure S1. — Flow chart of patient selection. Figure S2 Forest plot of eight studies with at least ten events for TGFBR2 rs1367610. Figure S3a Linkage disequilibrium of five top single nucleotide polymorphisms (SNPs) in IL12B associated with estrogen receptor (ER)-negative patients with chemotherapy. Figure S3b linkage disequilibrium of seven top SNPs in HDAC9 associated with triple-negative patients with chemotherapy. Figure S4 UCSC browser graphic for TGFBR2 rs1367610. [file 13058_2015_522_MOESM1_ESM.zip › 4888551391406903_add1/Figure S2.tiff]

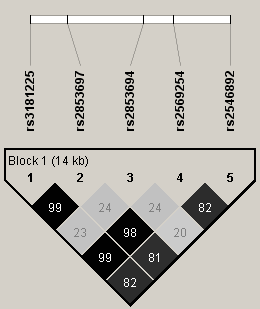

Supplement: Additional file 1: Figure S1. — Flow chart of patient selection. Figure S2 Forest plot of eight studies with at least ten events for TGFBR2 rs1367610. Figure S3a Linkage disequilibrium of five top single nucleotide polymorphisms (SNPs) in IL12B associated with estrogen receptor (ER)-negative patients with chemotherapy. Figure S3b linkage disequilibrium of seven top SNPs in HDAC9 associated with triple-negative patients with chemotherapy. Figure S4 UCSC browser graphic for TGFBR2 rs1367610. [file 13058_2015_522_MOESM1_ESM.zip › 4888551391406903_add1/Figure S3.tiff]

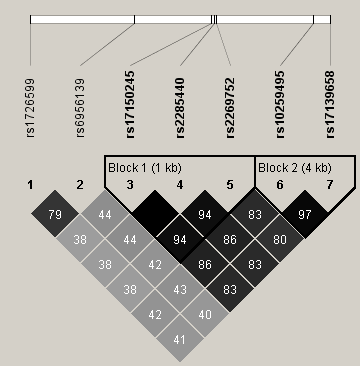

Supplement: Additional file 1: Figure S1. — Flow chart of patient selection. Figure S2 Forest plot of eight studies with at least ten events for TGFBR2 rs1367610. Figure S3a Linkage disequilibrium of five top single nucleotide polymorphisms (SNPs) in IL12B associated with estrogen receptor (ER)-negative patients with chemotherapy. Figure S3b linkage disequilibrium of seven top SNPs in HDAC9 associated with triple-negative patients with chemotherapy. Figure S4 UCSC browser graphic for TGFBR2 rs1367610. [file 13058_2015_522_MOESM1_ESM.zip › 4888551391406903_add1/Figure S4.tiff]

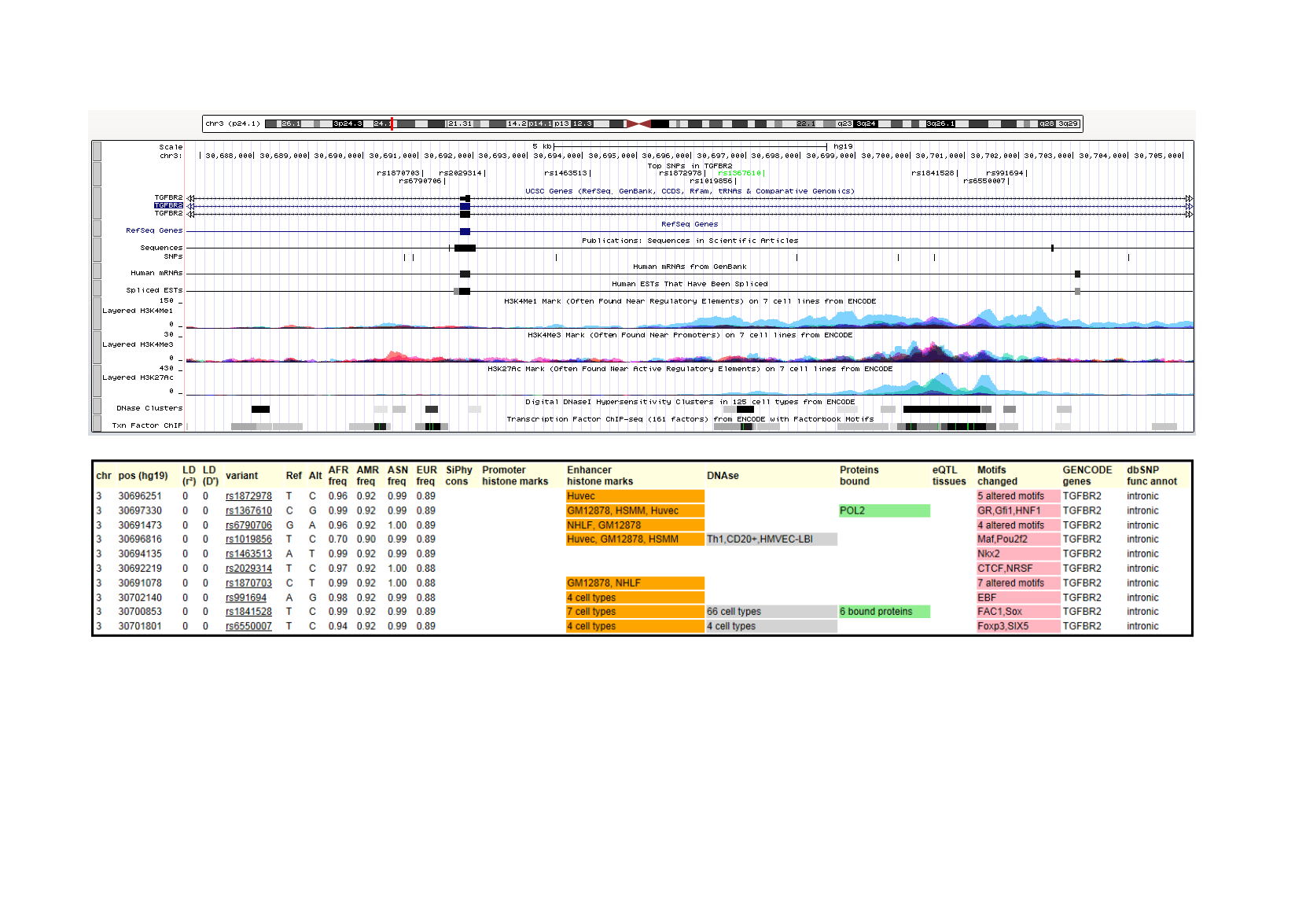

Supplement: Additional file 1: Figure S1. — Flow chart of patient selection. Figure S2 Forest plot of eight studies with at least ten events for TGFBR2 rs1367610. Figure S3a Linkage disequilibrium of five top single nucleotide polymorphisms (SNPs) in IL12B associated with estrogen receptor (ER)-negative patients with chemotherapy. Figure S3b linkage disequilibrium of seven top SNPs in HDAC9 associated with triple-negative patients with chemotherapy. Figure S4 UCSC browser graphic for TGFBR2 rs1367610. [file 13058_2015_522_MOESM1_ESM.zip › 4888551391406903_add1/Figure S5.tiff]
